# Mindfulness-based online intervention on mental health among undergraduate nursing students during coronavirus disease 2019 pandemic in Beijing, China: A randomized controlled trial

**DOI:** 10.3389/fpsyt.2022.949477

**Published:** 2022-11-16

**Authors:** Zhenwei Dai, Shu Jing, Hao Wang, Weijun Xiao, Yiman Huang, Xu Chen, Jiaqi Fu, Chen Pan, Qiuping Tang, Huan Wang, Xiaoyou Su

**Affiliations:** ^1^Department of Infectious Diseases, School of Population Medicine and Public Health, Chinese Academy of Medical Sciences & Peking Union Medical College, Beijing, China; ^2^Department of Clinical Psychology, The Third Xiangya Hospital of Central South University, Changsha, China; ^3^School of Nursing, Chinese Academy of Medical Sciences & Peking Union Medical College, Beijing, China

**Keywords:** nursing student, mindfulness, mental health, COVID-19, intervention

## Abstract

**Objective:**

To evaluate the effect of mindfulness intervention on improving mental health among undergraduate nursing students during the coronavirus disease 2019 (COVID-19) pandemic in China.

**Methods:**

An online mindfulness intervention course named Mindfulness Living With Challenge (MLWC) was developed by the research team, and a randomized controlled trial using MLWC among Chinese undergraduate nursing students was carried out. A total of 120 undergraduate nursing students were randomized into control (60 students) and intervention groups (60 students) *via* a WeChat mini program. Self-administered questionnaire surveys were conducted at pre- and post-intervention, measuring depression, anxiety, stress, mindfulness, and perceived social support. After intervention, the acceptance of the online mindfulness course among participants was assessed by employing the theory of technology acceptance model (TAM).

**Results:**

Among the enrolled 120 participants, 86.67% (52/60) and 93.33% (56/60) of the intervention and control groups remained completed the trial and the questionnaire surveys. Compared with the control group, the reduction of anxiety and stress symptoms, as well as the improvement of mindfulness level and perceived social support in the MLWC intervention group were statistically significant (*P* < 0.05), while the change in depression was not statistically significant. The scores of the four dimensions in TAM ranged from (5.88 ± 0.94) to (5.91 ± 0.97).

**Conclusion:**

Online mindfulness intervention implemented in this study is effective in improving mental health among undergraduate nursing students, and they were interested in this intervention.

**Clinical trial registration:**

[www.ClinicalTrials.gov], identifier [ChiCTR2 200058103].

## Introduction

The coronavirus disease 2019 (COVID-19) has posed a hazard to public health worldwide since its outbreak (1, 2). Due to lockdowns, isolation and decreased social interactions, etc., the COVID-19 pandemic also affected the mental health of people, especially among the youth (3). University students constitute a large population for the prevalence of mental disorders (4). A meta-analysis showed that during COVID-19, the prevalence of anxiety, depression, and sleep disorders among college students worldwide was 28, 31, and 40%, respectively, higher than that of other age groups (5). The prevalence of anxiety, depression, and sleep disorders among Chinese college students during the pandemic was 24, 23, and 24%, respectively, which were a bit lower than those of the global average. However, it is still far higher than the historical data in China (6, 7).

Mental illness of medical students has attracted widespread concerns in recent decades, especially after COVID-19, since it often has serious consequences among this population and their future patients (8). Overwhelming academic pressure, worries about the pandemic, insufficient physical activity, and excessive use of social media during the COVID-19 pandemic might be the major sources of the increasing rate of mental illness among medical students (9, 10). Undergraduate nursing students face the same stressors with their non-nursing peers in medical school, additionally, they have easier access to information about the pandemic and are more likely to develop mental disorders like depression, stress, and anxiety due to the fear of uncontrollability of the pandemic compared with those of other majors (11). During the COVID-19 pandemic, nurses have more chances to come into touch with patients and their body fluids, and thus increasing the risk of infection. Furthermore, the high working load and pressure confronted by the nurses during the pandemic may lead to certain mental illnesses for nursing students after learning about these conditions (12, 13). Moreover, they may also experience additional challenges associated with mandatory clinical practicum and social prejudice from family and friends due to their future occupational identity as a nurse (14, 15). A cross-sectional study on the mental health of Chinese undergraduate nursing students during the COVID-19 pandemic showed that the prevalence of anxiety and depression was 55.0 and 56.4%, while 31.6% of nursing students suffered from the two disorders simultaneously, which was higher than those among other medical students (16). According to the previous studies, mental disorders could have substantial negative impact on undergraduate nursing students, and those affected by mental disorders are more likely to have psychosomatic problems like insomnia, interpersonal difficulties, and low learning efficiency (17–19).

The COVID-19 pandemic has witnessed a severe shortage of nurses in China, which has hampered the development of overall population health and revealed the country’s weaknesses in health system (20, 21). Undergraduate nursing students constitute the main force of nursing profession in the future. However, the mental health challenges they face are directly associated with their self-growth and career trajectory, which could further impact the overall wellbeing of the nursing profession in China (22). Therefore, more attention should be paid to helping undergraduate nursing students improve their psychological wellbeing.

Mindfulness is a psychological trait related to attention and awareness (23). It can be defined as an open and conscious observation of one’s present experience, which is characterized by non-judgmental awareness of the present experience, including awareness of individual’s feelings, thoughts, physical state, consciousness, and environment, while encouraging openness, curiosity, and acceptance (23). Mindfulness intervention is aimed at enhancing the awareness of the present experience, alleviating negative emotions, and improving the quality of daily life (24, 25). Mindfulness-based intervention is an easy-to-transmit and non-invasive psychological intervention method, which has been proved to have remarkable effect on reducing mental disorders (26). Mindfulness intervention has also been proven to be effective in improving the mental health of undergraduate nursing students in the US, the UK, and Singapore (27–30). Besides, internet-based mindfulness intervention has also been shown to be effective in prevention of mental disorders in various populations, and it could provide a low-cost and convenient way to make intervention more accessible to people who would otherwise be excluded (31).

In China, some scholars have introduced mindfulness intervention to college students, while few studies have been conducted to evaluate the effect of online mindfulness intervention on undergraduate nursing students. A randomized controlled trial reported that mindfulness intervention could effectively reduce anxiety symptoms of Chinese nursing students, but this study was an offline intervention conducted before the COVID-19 pandemic and the intervention skipped some core practice of mindfulness such as mindfulness stretching and mindfulness awareness practice due to feasibility consideration (32). The current study aims to explore the effect of an online mindfulness-based psychological intervention on the mental health of nursing undergraduates during COVID-19. In this study, we used the online course Mindfulness Living With Challenge (MLWC) developed by our research team to conduct a 6-week mindfulness intervention among undergraduate nursing students. The MLWC intervention course incorporates mindfulness meditation and mindful stretching as core components, as well as some elements from Mindfulness-Based Stress Reduction (MBSR) and Mindful Awareness Practices (MAP). A similar intervention named Mindfulness Living With Stress (MLWS) has been successfully implemented among nurses in China (33). The research team has also proposed a protocol for the rehabilitation of mental health of COVID-19 survivors using MLWC (34). The current study is aimed at evaluating the effects of the online MLWC intervention on depression, anxiety, stress, mindfulness level, and perceived social support of undergraduate nursing students *via* WeChat mini program developed by the research team, and assessing the acceptance of online MLWC intervention *via* mini program based on technology acceptance model (TAM), to provide reference for further research and intervention on improving psychological wellbeing of undergraduate nursing students.

## Materials and methods

### Trial design

**FIGURE 1:**
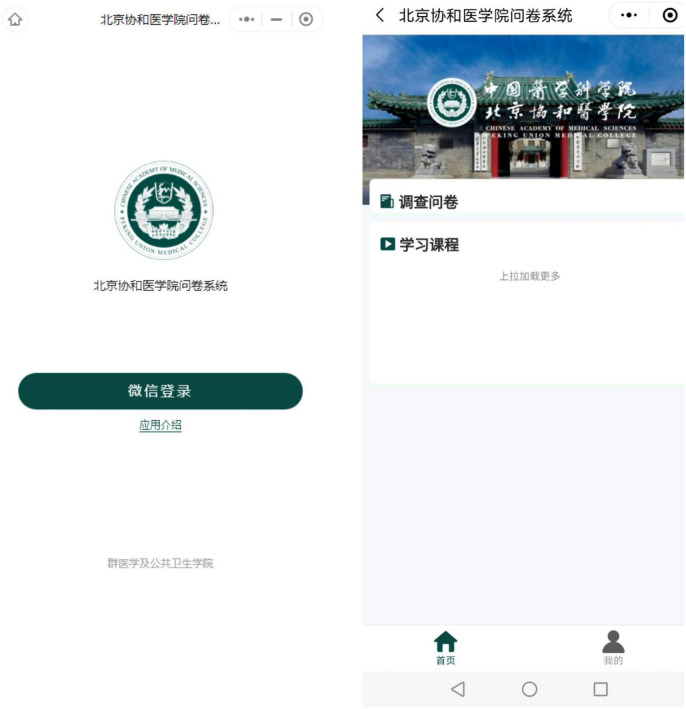
Interfaces of “Peking Union Medical College Questionnaire System” mini program.

This is a randomized, controlled, open-label online mindfulness intervention study. We first conducted a baseline survey among 240 undergraduate nursing students who volunteered to participate in this study, after that, we used a random digit to select 120 undergraduate nursing students from the baseline survey to be included in the randomized controlled trial. The researchers entered the basic information of the participants into the “Peking Union Medical College Questionnaire Survey” (PUMCQS) WeChat mini program that was specifically designed to distribute questionnaires and intervention materials (Figure 1), and uniformly registered their login identities. Based on a random digit generated by PUMCQS, 120 participants were randomly assigned to MLWC intervention group and waitlist control group, with 60 students in each group. The intervention group received mindfulness training and the control group received health education unrelated to the content of the intervention material. Since the mindfulness course requires the students of intervention group to participate in and receive training, this study is an open-label trial, which does not involve blind methods (35). MLWC, the online intervention course used in this study, was developed by two Chinese psychiatrists, one of whom has been certified in the Training of Mindfulness Facilitation program at the Mindful Awareness Research Center of University of California, Los Angeles (UCLA). The course has obtained a work registration certificate from the Copyright Administration of the People’s Republic of China (registration number: GZDZ-2021-I-00193781). The study was approved by the institutional review board of the investigators’ university (ID: CAMS&PUMC-IEC-2021-009).

### Participants

#### Sample size

To achieve 80% power to reject the null hypothesis of equal means when the mean difference of depression score is 5.0 with a standard deviation for both groups of 9.0 and with a significance level of 0.050 using a two-sided two-sample equal-variance *t*-test, group sample sizes of 52 and 52 for intervention group and control group are needed.

#### Participants recruitment

Participants were recruited from a school of nursing at a medical university in Beijing, China through phone, posters, and online forums from 25 to 30 April 2021. The following were eligibility criteria: (1) Undergraduate nursing students over 18 years old, (2) Be able to work independently with investigators to complete the questionnaire survey, (3) Have mobile communication equipment such as a mobile phone, and a WeChat account, (4) Mobile equipment can access the internet at any time, and (5) Have not received mindfulness intervention within 6 months prior to enrollment in the study. In total, 120 undergraduate nursing students signed up for the program and gave their informed consent.

### Intervention and implementation

**FIGURE 2:**
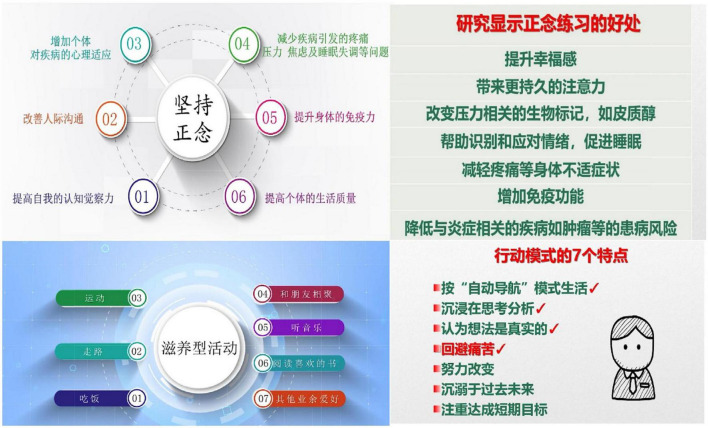
Mindfulness living with challenge lesson content.

Participants assigned to the MLWC intervention group were asked to complete a 6-week mindfulness training program that included six sessions (two lessons per session) and offered from 10 May 2021 to 20 June 2021. During the course, participants were required to learn and practice 2 days a week and 30–40 min for each day, including theoretical instruments, meditation practices, and homework in the form of audio and video lessons. The procedure was as follows: investigators invited participants of MLWC intervention group to an auditorium 2 days a week and played one MLWC lesson on the projector screen each time. The mindfulness course includes video and audio of mindfulness meditation, mindfulness stretching, mindfulness walking, etc. During each course, the research team members participated in the whole process and were responsible for Broadcasting the MLWC course in an auditorium, and supervising the learning and practice of the intervention group. The contents of MLWC are shown in Table 1 and Figure 2. Considering the limited space of the auditorium for practice, after each lesson, participants were required to log into the WeChat mini program. The meditation practice and homework section of each lesson were uploaded on the mini program for them to practice before the next lesson. All materials were presented in Chinese, which is the native language for all participants. To enhance adherence, push notifications from the WeChat mini program were sent to the participants every Friday to remind them of their weekly attendance and regular practice. Researchers could also check the completion of practice at the console of the mini program.

**TABLE 1:** The content of mindful living with challenge (MLWC) intervention.

Week	Practice	Content	Homework
1 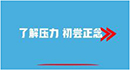	Mindful eating practice, mindful breathing meditation	Introduction to mindfulness and its importance. Summaries on how to incorporate mindfulness into daily life, previous application and scientific findings of mindfulness-based interventions.	Daily life mindfulness practices and mindful breathing meditation for 5–10 min daily for 7 days a week.
2 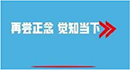	Mindful body scan practice, 3-min breathing space practice	Introduction to brain’s mode of action and being. Instructing participants to talk to their bodies	Mindful body scan practice, 3-min breathing space practice, and filling in pleasant/unpleasant experiences calendar for 10–15 min daily for 7 days a week, respectively.
3 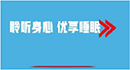	Sitting meditation (mountain meditation and mindful sleep meditation)	Introduction to the scientific understanding of sleep, sleep hygiene education, and seven attitudes of practicing mindfulness	Sitting meditation and mindfulness clock for 15 min daily for 7 days a week, respectively
4 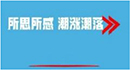	Mindful walking meditation, sitting meditation (lake meditation)	Introduction to mindful living with thoughts, using the “STOP” and “RAIN” principle to deal with a storm of thoughts and emotions	Mindfulness listening practice, mindfulness movement, and STOP/RAIN practice for 15–20 min daily for 7 days a week, respectively
5 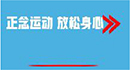	Mindful yoga practice	Introduction to mindful movement for relaxing body and mind. Explanation of identification of avoidance response, allowing and letting it go	Sitting meditation for 15 min daily for 7 days a week and practice mindful yoga three times a day as appropriate
6 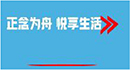	Sounding meditation; loving and kindness meditation	Introduce to tired funnel and how to balance daily life, mindful living with a challenge, and how to live a mindful life, live in the present	listing nourishing/consuming activities and weaving mindfulness parachute

### Outcomes

#### Primary outcomes: Depression, anxiety, and stress

Depression-Anxiety-Stress Scale (DASS-21) was used to assess participants’ mental health in the past 2 weeks (36). It consists of 21 items and allows for the simultaneous assessment of three psychological problems: depression, anxiety, and stress. Each item is rated on a 4-Likert scale from 0 to 3, with higher scores indicating more severe psychological problems. The total scores of depression ≥ 5, anxiety ≥ 4 and stress ≥ 7.5 indicate corresponding symptoms. The scale has been verified among Chinese college students and showed good reliability and validity (37). Cronbach’s alpha of DASS-21 was 0.869 at baseline, and 0.748 at post-assessment. KMO value was 0.771 at baseline, and 0.731 at post assessment, indicating good reliability and structural validity in this study.

#### Secondary outcomes: Mindfulness and perceived social support

The mindfulness level of participants in the past 2 weeks was measured by Chinese Short Formed Five Facets of Mindfulness Questionnaire (FFMQ-SF) (38, 39). The FFMQ-SF consists of 20 items which are rated on a 5-point Likert scale. It measures five facets of mindfulness: observing, describing, acting with awareness, non-judging, and non-reacting. The items of “acting with awareness” and “non-judging” factors are reverse scored. A higher total score on the 20 items indicates a higher level of mindfulness. The scale has been verified to be applicable to Chinese people with acceptable reliability and validity (40). Cronbach’s alpha of FFMQ-SF was 0.817 at baseline, and 0.710 at post-assessment. KMO value was 0.813 at baseline, and 0.800 at post assessment, indicating good reliability and structural validity in this study.

The Perceived Social Support Scale (PSSS) was used to assess participants’ perceived social support in the past 2 weeks from three sources: family, friends, and significant others (41). It consists of 12 items which are rated on a 7-point Likert scale. A higher total score indicates more perceived social support. The scale shows satisfied validity and reliability among Chinese college students (42). Cronbach’s alpha was 0.961 at baseline, and 0.947 at post-assessment. KMO value was 0.933 at baseline, and 0.917 at post assessment, indicating good reliability and structural validity in this study.

#### Acceptance of online mindfulness course *via* mini program

The acceptance of the online mindfulness course *via* mini program of intervention group was assessed using the theory of TAM from 4 dimensions: perceived ease of use, perceived usefulness, attitude, and behavioral intention (43). The instrument consists of 20 items, and each item is 7-point Likert scaled. Higher scores indicate higher acceptance of the online course *via* mini program. The Cronbach’s alpha of this instrument employed to intervention group was 0.991. KMO value was 0.821, indicating good reliability and structural validity in this study.

### Statistical analysis

Intention-to-treat analysis was used as a stringent test of efficacy. Independent sample *t*-test and chi-square test were used to examine the comparability of baseline demographic and psychological variables between the intervention group and waitlist control group. An independent sample *t*-test was applied to test the differential value of psychological variables of post-assessment minus baseline assessment between intervention group and waitlist control group using SAS 9.4. According to the recommendation from literatures, partial least squares structural equation modeling (PLS-SEM) was employed for assessing the TAM in this study, since this method is best suitable for building structural equation model with small sample size (44). The average variance extracted (AVE) and Composite Reliability (CR) were used to evaluate the measurement model. The coefficient of determination (*R*^2^), Stone-Geisser’s *Q*^2^-value, effect size *f*^2^-value, and path coefficients were employed to evaluate the structural model (45). The bootstrap analysis was performed to test the significance of the path in the model with 5,000 bootstrap samples at the level of α = 0.05.

## Results

### Demographic characteristics

**FIGURE 3:**
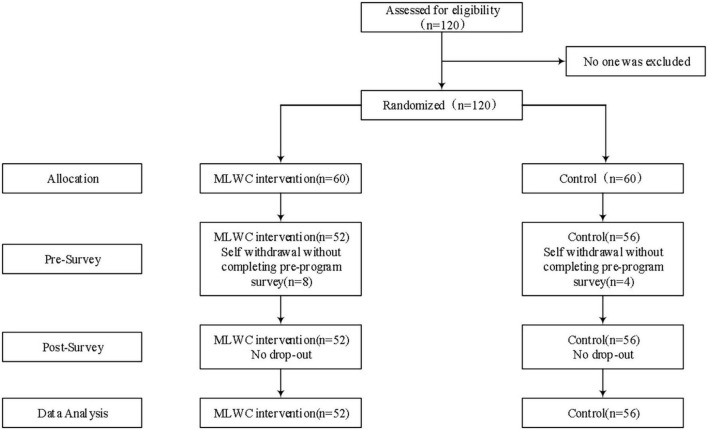
Flowchart of the randomized controlled trial. The coefficients are all standardized and statistically significant at the level of α = 0.05; USEFUL, Perceived ease of use; Easy to Use, Perceived ease of use; ATT, Attitude; BI, Behavioral intention.

Among the enrolled 120 participants, 6 participants in the MLWC intervention group and 4 participants in the waitlist control group refused to continue to participate in the study after they were assigned in this trial. Finally, 86.67% (52/60) and 93.33% (56/60) of the intervention and control groups were included in the trial. The study participants had a mean age of 19.16 (*SD* 0.80); the majority (86/108, 79.63%) was female; the prevalence of depression, anxiety, and stress was 24.1, 23.1, and 11.1%, respectively. None of them reported previous experience on systematic mindfulness and related practice. Two independent sample *t*-test and Chi-square analysis revealed no cross-group differences in participants’ demographic characteristics. The retention rate at post-program was 100% (see Figure 3). The demographic characteristics of the participants are shown in Table 2.

**TABLE 2:** Demographic characteristics of participants.

	MLWC (*n* = 52)	Control (*n* = 56)	Statistics	*P*
Age	19.31 ± 0.85	19.02 ± 0.73	1.895	0.061
**Gender**			0.453	0.501
Male	12 (23.1%)	10 (17.9%)		
Female	40 (76.9%)	46 (82.1%)		
**Have position in student union**			0.326	0.568
No	25 (48.1%)	30 (53.6%)		
Yes	27 (51.9%)	26 (46.4%)		
**Residence**			0.138	0.710
City	37 (71.2%)	38 (67.9%)		
Village	15 (28.8%)	18 (32.1%)		
**The only child in family**			1.931	0.165
Yes	32 (61.5%)	27 (48.2%)		
No	20 (38.5%)	29 (51.8%)		
**Father’s educational level**			4.277	0.378
Below junior high school	3 (5.8%)	0 (0.0%)		
Junior high school	15 (28.8%)	20 (35.7%)		
Technical secondary school	6 (11.5%)	9 (16.1%)		
Senior high school	7 (13.5%)	9 (16.1%)		
Undergraduate or above	21 (40.4%)	18 (32.1%)		
**Mother’s educational level**			1.353	0.884
Below junior high school	4 (7.7%)	5 (8.9%)		
Junior high school	16 (30.8%)	14 (25.0%)		
Technical secondary school	9 (17.3%)	14 (25.0%)		
Senior high school	10 (19.2%)	11 (19.6%)		
Undergraduate or above	13 (25.0%)	12 (21.4%)		
**Reason for selecting nursing major**			0.019	0.892
Own will	35 (67.3%)	37 (66.1%)		
Not own will	17 (32.7%)	19 (33.9%)		
**Have family members engaged in nursing**			1.700	0.192
Yes	7 (13.5%)	13 (23.2%)		
No	45 (86.5%)	43 (76.8%)		
**Depression symptom**			2.459	0.117
No	36 (69.2%)	46 (82.1%)		
Yes	16 (30.8%)	10 (17.9%)		
**Anxiety symptom**			1.830	0.176
No	37 (71.2%)	46 (82.1%)		
Yes	15 (28.8%)	10 (17.9%)		
**Stress symptom**			0.019	0.892
No	46 (88.5%)	50 (89.3%)		
Yes	6 (11.5%)	6 (10.7%)		

### The effect of mindfulness living with challenge intervention

Compared with the control group, the reduction of anxiety and stress symptoms, and the improvement of mindfulness level and perceived social support in MLWC intervention group were statistically significant (*P* < 0.05), while the change of depression was not statistically significant, which are displayed in Table 3.

**TABLE 3:** The effect of mindfulness training on mental health of participants.

	MLWC (*n* = 52)	Control (*n* = 56)	Statistics	*P*
**Depression**				
Baseline	2.44 (1.79, 3.09)	2.38 (1.80, 2.96)	0.152	0.880
Differential value	−0.83 (−1.50, −0.15)	−0.29 (−0.77, 0.19)	–1.303	0.195
**Anxiety**				
Baseline	2.48 (1.88, 3.08)	2.11 (1.62, 2.60)	0.952	0.343
Differential value	−1.25 (−1.82, −0.68)	0.00 (−0.46, 0.46)	–3.385	0.001
**Stress**				
Baseline	4.00 (2.90, 5.10)	3.20 (2.47, 3.93)	1.241	0.217
Differential value	−1.90 (−2.89, −0.91)	−0.09 (−0.66, 0.48)	–3.100	0.003
**Mindfulness level**				
Baseline	65.65 (62.95, 68.35)	65.45 (63.25, 67.65)	0.118	0.906
Differential value	3.54 (0.44, 6.64)	−2.39 (−4.99, 0.21)	2.884	0.005
**Perceived social support**				
Baseline	66.69 (62.59,70.79)	65.86 (62.46, 69.26)	0.309	0.758
Differential value	4.62 (1.42, 7.82)	−0.32 (−2.42, 1.78)	2.572	0.011

### Acceptance of mindfulness living with challenge intervention *via* mini program

**FIGURE 4:**
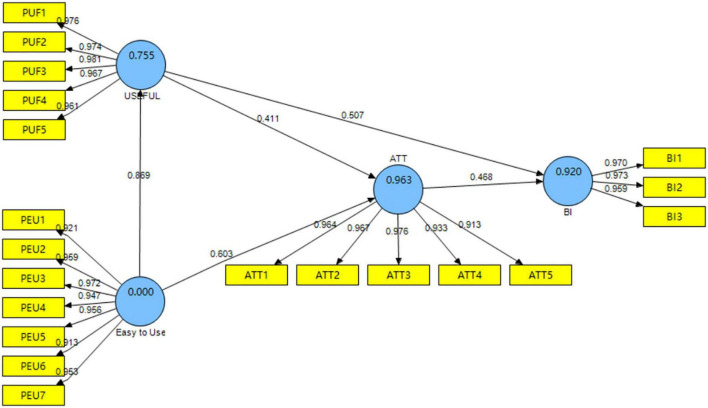
Technology acceptance model of MLWC intervention *via* mini program.

The scores of the 4 dimensions in TAM were from (5.88 ± 0.94) to (5.91 ± 0.97) (see Table 4). The CR and AVE value of the model were all above 0.7 and 0.5, respectively, indicating good validity of the model. The *R*^2^ and *Q*^2^ were all above 0.67 and 0, respectively, indicating high predictive power of the model (see Table 5). The result of path analysis showed that all the paths were statistically significant, and the *f*^2^ were all above 0.35, which indicated large effect of each path (see Table 6). The model built in the current study is illustrated in Figure 4.

**TABLE 4:** Mean scores of five dimensions in TAM model.

Dimension	Mean (*SD*)
Perceived ease of use	5.91 (0.97)
Perceived usefulness	5.85 (0.93)
Attitude	5.88 (0.94)
Behavioral intention	5.90 (0.97)
Total	5.89 (0.92)

**TABLE 5:** Evaluation of measurement model and predictive power.

Dimension	*R* ^2^	*Q* ^2^	CR	AVE
Perceived ease of use			0.984	0.895
Perceived usefulness	0.755	0.705	0.988	0.944
Attitude	0.963	0.859	0.979	0.904
Behavioral intention	0.920	0.849	0.978	0.936

**TABLE 6:** Path analysis and effect size.

Path	β	*T*	*P*	*f* ^2^
Attitude - > Behavioral intention	0.468	2.72	0.007	0.342
Perceived ease of use - > attitude	0.603	8.578	0.000	2.41
Perceived ease of use - > perceived usefulness	0.869	21.702	0.000	3.086
Perceived usefulness - > behavioral intention	0.507	2.985	0.003	0.403
Perceived usefulness - > attitude	0.411	5.824	0.000	1.122

## Discussion

In this study, the rates of depression, anxiety, and stress among undergraduates were 24.1, 23.1, and 11.1%, respectively, all higher than the results of cross-sectional studies conducted among vocational nursing students in Henan and Sichuan, China during and before COVID-19 pandemic, respectively (46, 47). Greater academic pressure, decreased social interaction and physical activity caused by lockdown, and the perceived uncertainties of the COVID-19 have all increased the possibility of anxiety and depression among undergraduate nursing students (48, 49). In China, vocational nursing students account for the majority of nursing students at present, and many people think it is not necessary for nurses to have professional academic training due to the deep-rooted opinion on simplicity of nurses’ clinical work. Accordingly, undergraduate nursing students in China often suffer from incomprehension or even prejudice from their peers and other surrounding people, which could further lead to their poor mental health and failure in a smooth transition from student to registered nurse after graduation (50–52). Therefore, more importance should be attached to improving the mental health of undergraduate nursing students.

The findings of this study suggested that MLWC intervention could reduce the symptoms of anxiety and stress among undergraduate nursing students, which is in consistence with other research that applied MBSR to nursing students (53, 54). This finding indicated that educators might consider implementing MLWC interventions among undergraduate nursing students to alleviate their anxiety and stress symptoms. A systematic review showed that mindfulness intervention can improve individual’s mental health and lead to positive biomarker outcomes such as decreased interleukin-6 (IL-6) and tumor necrosis factor alpha (TNF-α) (55). A meta-analysis also suggested IL-6 and TNF-α may play a negative role in the pathogenesis of mental disorders (56). Additionally, a study on functional neuroimaging also showed that mindfulness training can cause morphological changes in the brain, such as decreased activation of the amygdala and increased activation of the prefrontal lobe, and these changes can help to alleviate negative emotions and reduce the interference of unpleasant stimuli on emotions (57). In this case, the positive effect of mindfulness intervention on anxiety and stress has its biological basis. However, in contradiction with some of the aforementioned studies, the current study indicated that MLWC intervention had no obvious effect on depression symptoms of undergraduate nursing students. Similarly, a study that applied an 8-week group mindfulness-based cognitive therapy to nursing students in Turkey observed a decrease in stress level but no significant change in depression level (58). Another study in Korea that employed a stress coping program based on mindfulness meditation displayed significant reduction of anxiety among nursing students, but had no significant effect on reduction of depression (59). This might be interpreted that the content of MLWC was mainly composed of MBSR and MAP, which put emphasis on the reduction of stress and anxiety, while interventions for depression were not specifically involved in this program. Moreover, anxiety and stress are more likely to be feelings of worry and insecurity caused by negative events, whereas depression is more likely to be influenced by multiple factors, including biomedical factors, which could be difficult to change with short-term psychological interventions (60, 61). Therefore, the effect of MLWC on depression of undergraduate nursing students is still to be explored in the long run.

The present study also indicated that MLWC has a positive effect on undergraduate nursing students’ mindfulness level and perceived social support level, which is in consistence with prior research findings (58, 62, 63). A core component of MLWC is mindfulness meditation, which could effectively improve individuals’ mindfulness level. Furthermore, undergraduate nursing students with a higher level of mindfulness might be more likely to be aware of their surroundings and hence accept different things at the present time. This awareness and acceptance could help them perceive support and recognition from their family, friends and colleagues easily (63, 64). This finding provides evidence on the effect of mindfulness on improving perceived social support among undergraduate nursing students in China. Educators could consider employing MLWC to undergraduate nursing students in order to enhance their perceived social support. Additionally, government could appeal to the society and general population to have more understanding of the nursing major and profession, reduce the prejudice against them, and provide more support for nursing students (14, 15).

The findings of the current study showed that TAM had a good fit to predict the acceptance of MLWC intervention *via* WeChat mini program among undergraduate nursing students. The scores of measurement model indicated that most of the participants believed the content and form of MLWC intervention were acceptable and helpful. Consequently, they held a positive attitude to MLWC intervention and were willing to introduce similar interventions to others. For this reason, nursing educators could consider referring to the results of this study and implementing online mindfulness intervention among undergraduate nursing students, given its high acceptance and usefulness in improving mental health. Furthermore, online intervention is also in accordance with the regulations on pandemic prevention and control during the COVID-19 pandemic.

Although the results of this study showed that MLWC has a positive impact in reducing mental disorders in undergraduate nursing students, it also has certain limitations. First, this study only recruited undergraduate nursing students from one university in Beijing, which may limit the generalization of the results; second, the sample size of 108 is relatively small, which might decrease the statistical power of this study; third, this study was quantitative without considering the qualitative evaluation on the experience of MLWC intervention among participants.

## Conclusion

Online MLWC intervention can improve the mental health of undergraduate nursing students in China during the COVID-19 pandemic. Nursing educators could consider implementing online MLWC intervention among nursing students based on the results of the present study. Further research could be planned on multicenter trials to confirm the effect of MLWC intervention on undergraduate nursing students and other populations.

## Data availability statement

Publicly available datasets were analyzed in this study. These data can be found here: http://www.medresman.org.cn/uc/projectsh/projectlistauthor.aspx.

## Ethics statement

The studies involving human participants were reviewed and approved by the Institutional Review Board of Chinese Academy of Medical Sciences. The patients/participants provided their written informed consent to participate in this study.

## Author contributions

XS, ZD, and SJ prepared the first draft and revised it critically for important intellectual content. XS and HuW provided overall guidance and managed the overall project. ZD, HaW, WX, YH, XC, JF, CP, QT, and SJ were responsible for the questionnaire survey, intervention implementation, and data analysis. All authors contributed to the article and approved the submitted version.
